# The Price of a Life: Unveiling the Struggle of Living With Hereditary Angioedema

**DOI:** 10.7759/cureus.42699

**Published:** 2023-07-30

**Authors:** Nicole L Welch, Joshua A Peterson, Kaka Adams

**Affiliations:** 1 Internal Medicine, Texas Tech University Health Sciences Center, Lubbock, USA; 2 Pediatrics, Texas Tech University Health Sciences Center, Lubbock, USA

**Keywords:** cocaine, angioedema management, immunology, c1 esterase inhibitor deficiency, medication affordability, hereditary angioedema

## Abstract

Hereditary angioedema (HAE) is a rare, potentially life-threatening genetic condition characterized by recurrent episodes of localized swelling in various body tissues. Despite advancements in the management and prevention of HAE, high costs limit accessibility to these medications and remain a significant hurdle for many patients. This case report illustrates the implications and life-threatening consequences of the affordability crisis associated with HAE medications. To the authors' knowledge, this case also highlights the first reported case of cocaine serving as an HAE trigger.

## Introduction

Hereditary angioedema (HAE) is a rare, autosomal dominant genetic disorder characterized by recurrent episodes of localized swelling in various body tissues [1]. It is caused by mutations in the C1 inhibitor gene, leading to dysregulation of the complement and contact systems [1]. This results in excessive bradykinin production and increased vascular permeability leading to subcutaneous and cutaneous edema [1,2]. 

Despite advances in understanding HAE pathophysiology and the development of effective treatment and prevention strategies, the management of this condition remains a challenge due to the high cost that limits access to these medications for some patients [3]. Through a detailed case report, we aim to shed light on the struggles faced by individuals with HAE and their difficulty in affording life-saving medications. 

Several studies have documented the financial challenges faced by individuals with HAE. Banerji et al*.* discussed the economic burden of HAE (including hospital, prescription, and other medical expenses incurred, lost wages, etc.) with the average annual cost per patient being an average of $42,000 to upwards of $96,000 for severe attacks with hospitalization and emergency department visits [3,4]. Castaldo et al. suggested that the average annual cost of on-demand-only treatment was as high as $363,795 [5]. These exorbitant financial impacts result in approximately 76% of patients reporting difficulty in affording HAE medications [6]. Another study found that physicians consider insurance coverage and cost as the main factors affecting their medication choices for HAE treatment and prophylaxis [7]. The financial burden of HAE medications places patients in a difficult situation as the consequences of untreated or poorly managed HAE attacks can be severe, resulting in increased healthcare utilization, hospitalization, and intensive care unit (ICU) stays. 

Addressing medication affordability in HAE is an issue that requires a multifaceted approach involving regulatory bodies, healthcare providers, pharmaceutical manufacturers, and policymakers. Increasing competition among manufacturers, price negotiations, and exploring alternative treatment options may help reduce the financial burden placed on individuals with HAE. This case report highlights a situation where the impact of medication affordability played a role in multiple intensive care unit admissions in a relatively short time frame. Secondarily, the patient’s HAE episodes detailed here were likely related to cocaine use, making this the first reported case of cocaine inducing HAE episodes.

## Case presentation

A 47-year-old male with a known medical history of hereditary angioedema (diagnosed 16 years ago) presented to the emergency department for facial swelling. He reported left eye swelling that began the previous evening. When he awoke the following morning, it had progressed to involve both eyes, the philtrum, and the upper lip. He denied taking any medications the previous evening and mentioned that he couldn't locate his epinephrine auto-injector, which he failed to use during the episode. He reported using epinephrine in the past without any relief of his symptoms. He denied any recent insect bites or consumption of new foods. He also recalled experiencing similar episodes in the past, requiring intubation three times previously due to angioedema, with the most recent episode occurring four months earlier.

**Figure 1:**
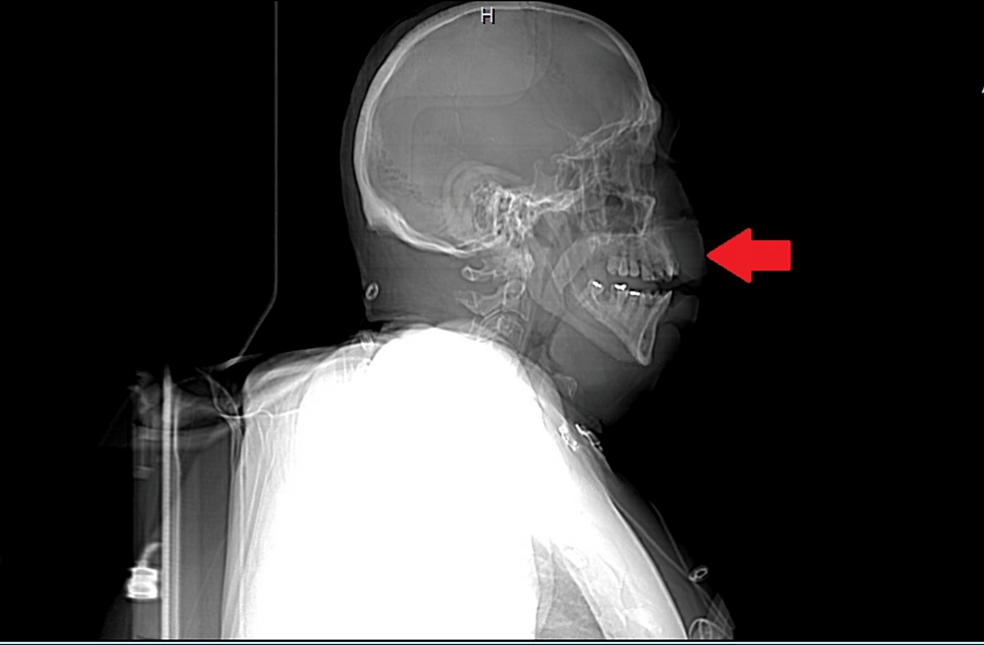
CT neck sagittal view showing angioedema of upper lip. The red arrow highlights the edema present in the upper lip.

**Figure 2:**
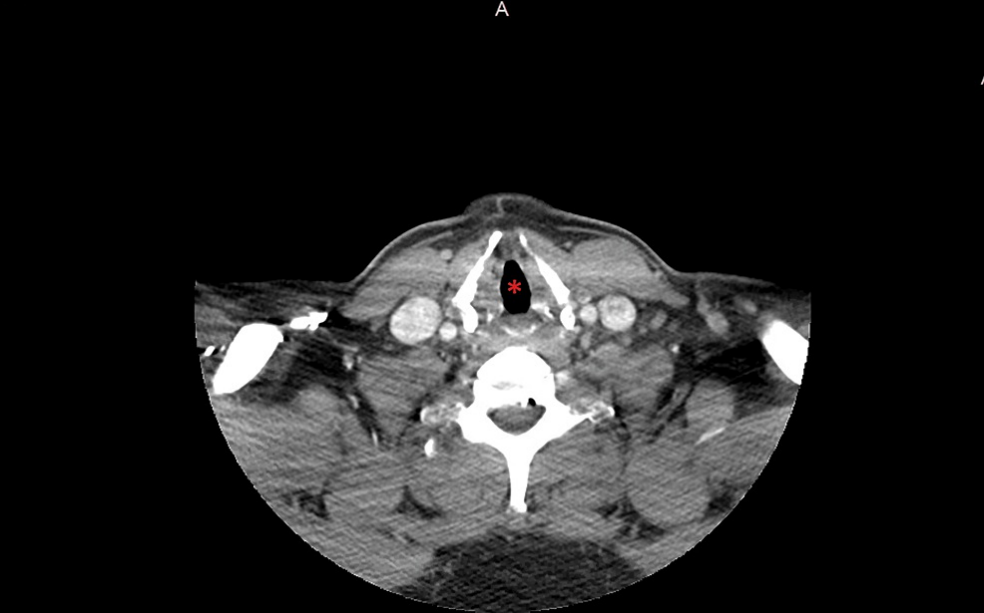
CT neck showing the patient's airway. The red asterisk highlights a patent airway, with no edema or airway compromise.

**Figure 3:**
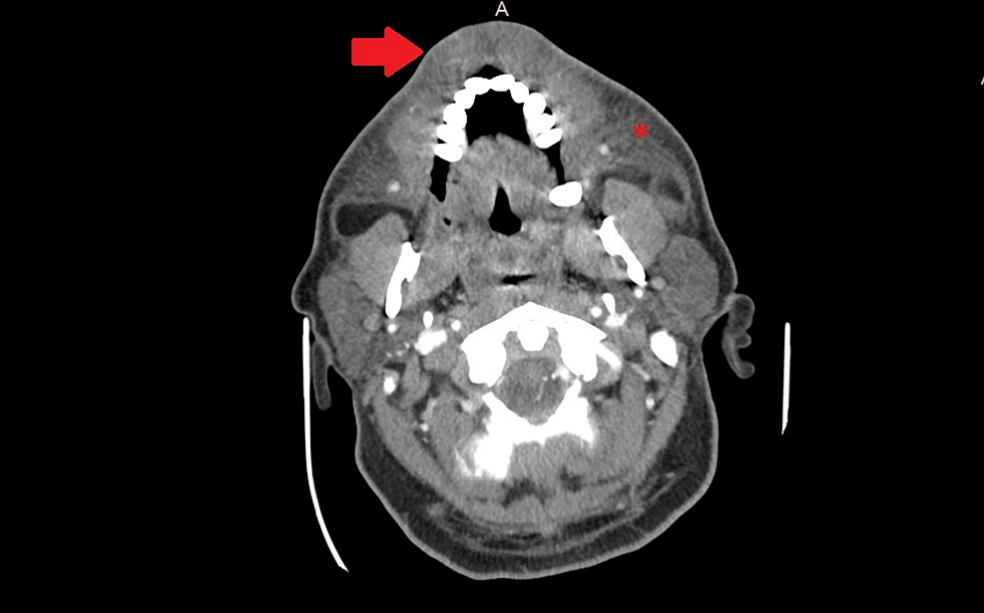
CT neck axial view highlighting subcutaneous edema. The red arrow shows massive enlargement of the upper lip while the asterisk highlights the diffuse involvement of the premaxillary soft tissues.

Emergency Medical Services administered dexamethasone, diphenhydramine, and famotidine to the patient prior to his arrival at the hospital. In the emergency department, the patient received a single dose of tranexamic acid, a controversial treatment option for acute attacks of hereditary angioedema. Laboratory tests yielded unremarkable results, indicating no significant abnormalities except for a urine drug screen positive for cocaine, low C4, and a C1 esterase inhibitor activity of 100% (Table 1). However, a computed tomography (CT) scan of the neck, shown in Figures 1-3, revealed striking superficial subcutaneous edema, with no evidence of airway compromise.

**Table 1:** Laboratory values on admission to the medical intensive care unit.

Lab	Result	Normal Range
White Blood Cell	9.89	4.23-9.07 K/uL
Hemoglobin	15.9	13.7-17.9 g/dL
Hematocrit	48.6	40.1-51.0%
Platelet	207	163-377 K/uL
Sodium	140	136-145 mmol/L
Potassium	4.0	3.5-5.1 mmol/L
Chloride	107	97-107 mmol/L
Bicarbonate	22	20-30 mmol/L
Glucose	95	65-115 mg/dL
Blood Urea Nitrogen	13	6-20 mg/dL
Creatinine	0.8	0.5-1.2 mg/dL
Complement 4	3	10-40 mg/dL
C1 esterase activity level	100	67-100%
Urine Drug Screen	Cocaine	negative

The patient was admitted to the intensive care unit (ICU) for close monitoring and observation. His condition improved and he was subsequently discharged the following day with danazol 200 mg twice daily for prophylaxis. A follow-up appointment with the allergy/immunology department was scheduled for two weeks later. However, the patient was unable to attend the appointment, as he returned to the emergency department with symptoms of shortness of breath and stridor on the day of his scheduled appointment. He required immediate intubation and as a result, did not receive a dose of C1 esterase inhibitor in the emergency room. He was again transferred to the ICU and extubated the following day. Labs again were significant for only a positive urine drug screen for cocaine and this time also cannabinoids. The patient was screened for Helicobacter pylori, a potential trigger for angioedema, which was negative. 

Given the persistent positive results for cocaine in his urine drug screen, there was suspicion that cocaine may have been a contributing trigger and the patient was advised to abstain from further drug use. The patient was again discharged home with an allergy/immunology follow-up appointment and was again prescribed danazol 200 mg twice daily, while social services worked on the approval process for a free year of IV C1 esterase inhibitor through a county assistance program. Unfortunately, the patient did not attend his allergy/immunology outpatient follow-up appointment, despite qualifying for free care and medications through a county assistance program. Eight months later, he was again seen in the emergency department for another episode of shortness of breath and stridor. The patient was not intubated and there was no mention of any home medications in Emergency Department documentation, including the danazol or the IV C1 esterase for which he was previously approved.

## Discussion

HAE can be both a debilitating and life-threatening illness, with a prevalence of approximately 1:50,000 patients in the general population in the United States [1,7]. In HAE, the deficiency or dysfunction of C1-INH, a protein within the complement system, leads to uncontrolled activation of the early complement cascade and excessive production of inflammatory mediators [1]. This results in inflammation, local edema, and various symptoms throughout the body [1]. Additionally, C1-INH regulates coagulation factors and inhibits kallikrein [1,7]. When unchecked, this contributes to the excessive release of bradykinin, a potent mediator of HAE symptoms [1,7]. The binding of bradykinin to its receptors on endothelial cells leads to increased vascular permeability, vasodilation, and smooth muscle contraction, causing the characteristic manifestations of HAE [1,2,7]. The understanding of the pathogenesis of HAE has paved the way for the development of targeted therapies, including C1-INH replacement therapy and bradykinin receptor antagonists, which have shown efficacy in reducing the frequency and severity of HAE attacks [1,7].

The cost of medications, including C1-INH replacement therapy and prophylactic treatments, can be substantial and serve as a barrier to access and adherence for patients with limited financial resources. The economic burden of HAE has been demonstrated in studies, with the average annual cost per patient estimated to be $42,000, predominantly driven by hospitalizations and long-term disease management costs [3-5]. Addressing the affordability of HAE medications is crucial to ensure equitable access to necessary treatments for all patients.

Another notable aspect of this case is the patient's history of cocaine use and the urine drug screen that was positive for cocaine. Several cases of Quincke’s Disease (isolated angioedema of the uvula) induced by cocaine use have been documented [8-11], though to the authors' knowledge, none of the patients were subsequently diagnosed with HAE. Though HAE was not diagnosed, cocaine-induced angioedema in these patients lends support to the hypothesis that cocaine could be a potential angioedema trigger in our patient. Trauma as well as emotional stress have also been associated with an increased risk of angioedema episodes in individuals with HAE [7]. The inhalation of cocaine can not only damage the nasal mucosa and airways but dependence is also associated with a chronic stress state [12]. All of these factors raise concern that cocaine was a triggering factor for our patient’s HAE episodes, making this case the first known report of cocaine as an HAE trigger. For HAE management and the patient’s overall health, the patient was advised to abstain from cocaine and was offered resources to overcome substance use disorder.

Despite attempts to see the patient for outpatient allergy/immunology follow-up, and local programs that would have decreased the cost of care, the patient did not attend follow-up visits where HAE prophylactic medication would have been prescribed. We speculate that concomitant substance use disorder and the costs of HAE therapy, as well as other work and social factors may have played a role in his subsequent non-adherence to follow-up. Reflection on this case indicates the value of an inpatient allergy/immunology evaluation of patients with newly diagnosed HAE, multiple HAE flares in a short time frame, or patients that are otherwise at high risk of being lost to follow-up. 

Regrettably, the cost of essential medications for HAE presents a major obstacle for patients, particularly those without insurance coverage. The rarity of the condition, limited competition among manufacturers, and a small target population contribute to the high prices of these drugs. Consequently, many individuals with HAE face difficulty affording the medications necessary to effectively manage their condition. This financial burden significantly impacts their quality of life and forces them to make challenging decisions, such as prioritizing HAE treatment or meeting basic needs. As demonstrated by this case, the repercussions of untreated HAE can lead to repeated ICU admissions and the need for intubation.

Addressing this medication affordability crisis will require a multi-faceted approach, including increased competition among manufacturers to lower prices, and possibly even government interventions to help negotiate drug prices. The purpose of this case was to bring awareness to the high costs of life-saving medications in this small population and the need for more affordable options or patient assistance programs to assist patients in managing their condition. 

## Conclusions

The primary objective of presenting this case was to raise awareness about the exorbitant prices of life-saving HAE medications within this limited patient population and to emphasize the urgent need for more accessible alternatives or patient assistance programs. We also underscore the importance of inpatient allergy/immunology evaluation in certain patients that may be at high risk of being lost to follow-up. By highlighting these challenges, we hope to advocate for changes that will enhance affordability and ensure that individuals with HAE can access the medications they require without compromising their financial well-being. In addition to discussing HAE medication accessibility, we share the first reported case of HAE episodes triggered by cocaine use.
